# Thirty second chair stand test: Test–retest reliability, agreement and minimum detectable change in people with early‐stage knee osteoarthritis

**DOI:** 10.1002/pri.1957

**Published:** 2022-05-19

**Authors:** Stephen Gill, Rachael Hely, Richard S. Page, Andrew Hely, Benjamin Harrison, Steve Landers

**Affiliations:** ^1^ Barwon Medical Imaging, Barwon Health Geelong Australia; ^2^ GIRADI Research Institute Geelong Australia; ^3^ School of Medicine Deakin University Waurn Ponds Australia; ^4^ Barwon Centre for Orthopaedic Research & Education (B‐CORE) St John of God Hospital Geelong Australia

**Keywords:** 30 s chair stand test, clinimetrics, knee osteoarthritis, minimum detectable change, performance‐based measures, reliability

## Abstract

**Background and Purpose:**

To determine intra‐session test‐retest reliability, agreement and minimum detectable change (MDC) of the 30 CST across three tests in people with knee osteoarthritis (OA).

**Methods:**

A test–retest reliability study was performed with 93 people with mild radiological knee OA. Participants were asked to complete three attempts of the 30 CST 1–2 min apart according to a standardised protocol. Participants completed three attempts on two occasions: baseline and 6 months later. Change between tests within each session was assessed with ANOVA's and post‐hoc *t*‐tests. Reliability was assessed with intra‐class correlation coefficients (ICC_[2,1]_). Measurement error was expressed as MDC for an individual (MDC_ind_) and a group (MDC_group_). Floor effects were considered present if more than 15% of participants scored zero for a test.

**Results:**

Scores increased by 0.5 and 0.8 stands between the first and second test (*p* < 0.05) at the baseline and 6‐month assessments respectively, and then stabilised between the second the third tests at the baseline assessment (*p* > 0.05) or decreased (0.3 stands) at the 6‐month assessment (*p* < 0.05). Scores demonstrated excellent reliability (ICCs >0.9). MDC_ind_ was approximately 2.5 stands and MDC_group_ was 0.3–0.4 stands. No floor effects were apparent.

**Discussion:**

The 30CST demonstrated a practice effect between the first and second tests, which was no longer apparent by the third test. Despite this, scores demonstrated excellent intra‐session reliability. MDC estimates provide clinicians and researchers with the smallest change that can be detected by the instrument beyond measurement error for individuals and groups in community‐dwelling adults with knee OA.

## 1 INTRODUCTION

Physical function is a primary outcome measure in knee osteoarthritis (OA) research and clinical practice (Dobson et al., 2013). Physical function can be measured using participant's self‐report, such as with questionnaires, or with performance‐based tests, where the participant performs a physical test. OARSI consensus guidelines recommend that performance‐based tests should occur alongside self‐report measures; tests that reproduce functional tasks are especially informative (Dobson et al., 2013). Walking, stair climbing and sit‐to‐stand are important functional tasks and are particularly relevant for people with knee OA (Dobson et al., 2013).

Sit‐to‐stand performance can be measured with the chair stand test. Original versions of the chair stand test assessed the time to complete 5 or 10 stands (Csuka & McCarty, 1985; Guralnik et al., 1994); however, these tests can be difficult to complete for people with lower limb pathology, leading to floor effects (Jones et al., 1999). Subsequently, the 30 s chair stand test (30 CST) was introduced, which measures the number of stands a person can complete in 30 s (Jones et al., 1999).

The 30 CST is now included in OARSI's recommended set of performance‐based tests for people with knee OA (Dobson et al., 2013). Despite the increasing use of the test, its clinimetric properties have received relatively little research attention. Reliability and agreement between measurements is a fundamental clinimetric property and considers the extent to which measurements are consistent and free from error (Portney & Watkins, 2015). Reliability assesses the degree to which patients can be distinguished from each other, despite measurement error and is often expressed as the intraclass correlation co‐efficient (ICC) (Terwee et al., 2007). Agreement considers differences between scores on repeated measurements in stable conditions and assesses absolute measurement error (Terwee et al., 2007). Originally, Jones et al. found the 30 CST had good test–retest reliability (ICC = 0.89, 95% CI: 0.79–0.93) in community dwelling adults who did not have lower limb pain (Jones et al., 1999). Four studies have subsequently investigated test–retest reliability in people with knee OA and found high ICCs (≥0.90), but performance consistently improved during the second attempt (Gill & McBurney, 2008; Holm et al., 2021; Tolk et al., 2019; Unver et al., 2015). Because only two tests were completed in each study, it remains uncertain if scores stabilise at or after the second attempt; hence, further research using a minimum of three tests is required. Understanding score stability is important in clinical practice and research for attributing the extent to which observed change in performance should be attributed to measurement error or real change.

The current study aimed to determine test‐retest reliability, agreement and minimum detectable change (MDC) of the 30 CST across three tests completed 1–2 min apart in community‐dwelling adults with knee OA.

## 2 MATERIALS AND METHODS

### 2.1 Design

Prospective test‐retest reliability and agreement study.

### 2.2 Subjects

Ninety‐three people with knee OA who were participating in prospectively registered clinical trials were included in this reliability study (ANZCTR, http://www.anzctr.org.au/). Regarding sample size, we assumed at least 50 participants would be required to allow adequately precise reliability estimates (Hopkins, 2000). Full eligibility criteria are described elsewhere (Landers et al., 2017) but in summary, eligible participants were aged 18–75 years and had moderate to severe unilateral knee pain with grade 2 knee OA as per the Kellgren‐Lawrence Grading scale (Kellgren & Lawrence, 1957).

### 2.3 Procedure

**FIGURE 1:**
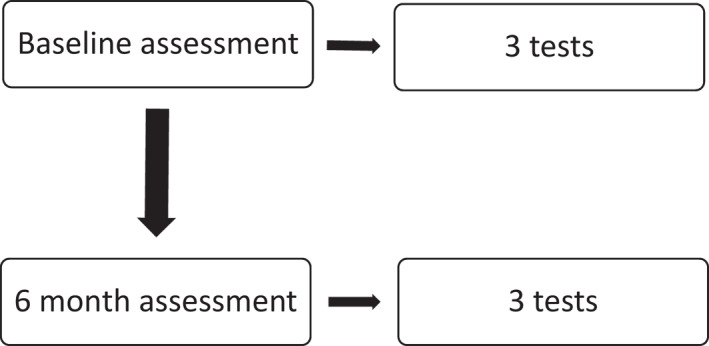
Study flow diagram: Each participant completed three tests, 6 months apart

The 30 CST procedure is described in Appendix  and was the same protocol as used in earlier research (Gill & McBurney, 2008). Participants were requested to complete as many sit to stand movements as they could from a chair in 30 s. All instructions, encouragement and measurements were conducted according to this standardised protocol. Within session reliability was assessed on two separate occasions, 6 months apart. At each assessment session, participants were asked to complete the test on three separate occasions approximately 1–2 min apart (Figure 1). The participant completed each subsequent test when at least 1 min had passed from the prior test and any fatigue or pain had subsided. Assessment sessions occurred at baseline and 6 months as part of the clinical trial that participants were involved in, which provided the opportunity to assess test‐retest reliability of two occasions.

One assessor administered all tests. The assessor was an Australian trained physiotherapist with 20 years of experience in assessing and treating musculoskeletal conditions and had experience administering the test as part of clinical practice prior to the study. The assessor was trained by the primary author to deliver the test according to the written standardised protocol.

### 2.4 Agreement between repeated tests

Several statistical approaches are available for determining reliability and agreement (Kottner et al., 2011). The most appropriate approach is debated and likely varies according to the specific study (Kottner et al., 2011). In the current study, to assess within session changes across the three tests, repeated‐measures analysis of variance (ANOVA) was conducted with post‐hoc *t*‐tests if a significant difference was found. As repeated‐measures ANOVA relies on the assumption of sphericity, adjustments were made when necessary (Field, 2005). Measurement error was expressed using the standard error of measurement (SEM), coefficient of variation (CV) and MDC. SEM was determined as the square root of the mean square error term in the repeated measures ANOVA (Atkinson & Nevill, 1998). The CV, or typical percentage error for the SEM, was expressed as the percentage of the mean score for the three tests (Hopkins, 2000). MDC was defined as the smallest change that can be detected by the instrument beyond measurement error (de Vet et al., 2006). To enable comparison with our previous research (Gill & McBurney, 2008), we expressed MDC as the amount of change required to be 90% confident that an observed change reflected real change rather than measurement error. MDC_ind_ equalled 1.65 × √2 × SEM, and represents the smallest detectable *within‐person* change for an individual (Terwee et al., 2007). MDC_group_ equalled MDC_ind_ divided by √*n*, and represents MDC for a group of people (Terwee et al., 2007).

#### 2.4.1 Reliability

Reliability was expressed with the (ICC_2,1_) and used a two‐way mixed effects model to assess multiple scores from the same assessor (Shrout & Fleiss, 1979). Each participant was assessed by the same rater, a physiotherapist, and we expect that the results can be generalised to other raters with similar characteristics (Portney & Watkins, 2015). We considered an ICC of less than 0.5 to represent poor reliability, 0.5–0.75 to represent moderate reliability, 0.76–0.90 to represent good reliability, and values above 0.90 to represent excellent reliability (Portney & Watkins, 2015).

Because agreement and reliability were assessed by comparing scores across three attempts, participants who could not complete all three tests due to exacerbation of knee pain were removed from this analysis.

Analysis was completed using SPSS Statistics for Windows (IBM Corp. Version 26.0).

### 2.5 Floor effects

Floor and ceiling effects are important measurement properties, and when present affect reliability (Terwee et al., 2007). Ceiling effects are not relevant for the 30 CST because the test has no maximum score. Floor effects were considered present if more than 15% of participants scored zero for a test (Terwee et al., 2007). The reason why a participant scored zero, such as intolerable knee pain, was also recorded.

### 2.6 Ethical considerations

Ethical approval was provided by the study organisation's Human Research Ethics Committee (refs: 15/101, 18/135) and all participants provided informed written consent.

## 3 RESULTS

### 3.1 Participants

Participant characteristics are provided in Table 1. In summary, participants were overweight or obese older adults who had experienced knee pain for an average of 3 years. All 93 participants were assessed at baseline, however due to COVID‐19 restrictions on social contact, 68 participants were assessed at 6 months.

**TABLE 1:** Participant characteristics at baseline assessment

Characteristic (*n* = 93^a^)	Summary statistic
Age, years; mean (SD)	61.3 (8.5)
Female; *n* (%)	55 (59.1%)
BMI; mean (SD)	33.5 (7.5)
Healthy weight^b^; *n* (%)	8 (8.6%)
Overweight^b^; *n* (%)	30 (32.2%)
Obese^b^; *n* (%)	55 (59.1%)
Symptom duration, years; mean (SD)	3.2 (4.1)
Knee pain intensity^c^; mean (SD)	45.9 (18.3)

Abbreviations: BMI, body mass index; SD, standard deviation.

^a^ Includes all participants who completed at least one attempt of the test.

^b^ Healthy weight (BMI 18.5–24.9), Overweight (BMI 25–29.9), Obese (BMI >30).

^c^ Knee Osteoarthritis Outcome Score Pain Scale (Roos et al., 1998), range 0–100 where 100 = no pain.

#### 3.1.1 Agreement and reliability

For participants who were able to complete all three tests, statistically significant improvements occurred between test 1 and 2 at baseline and 6‐month assessments (ANOVA *p* < 0.05); the difference was 0.5–0.8 stands respectively (Table 2). Performance stabilised between the second and third tests at the baseline and 6‐month assessment, however a statistically significant deterioration (0.25 stands) was still found at the baseline assessment between the second and third tests. MDC_ind_ was 2.3 and 2.8 stands, and MDC_group_ was 0.3 and 0.4 stands respectively at each assessment. ICC's exceeded 0.90 (Table 2). The coefficient of variation approached 10% at the baseline and 6 months assessment (Table 2).

**TABLE 2:** Agreement and reliability

	*N*	Test 1	Test 2	Test 3	Difference tests 1–2 (95% CI)	Difference tests 2–3 (95% CI)	SEM	CV	MDC_ind_	MDC_group_	ICC_(2,1)_ (95% CI)
Baseline	83^a^	9.64 (2.88)	10.16 (3.02)	9.90 (3.43)	0.52 (0.28,0.75)	−0.25 (−0.49, −0.02)	0.97	9.84%	2.27	0.25	0.92 (0.88, 0.94)
6 months	61^a^	12.15 (4.16)	12.93 (4.79)	13.03 (4.96)	0.79 (0.39, 1.18)	0.10 (−0.17, 0.37)	1.20	9.42%	2.79	0.36	0.94 (0.90, 0.96)

Abbreviations: ICC, intraclass correlation coefficient; CV, coefficient of variation; MDC_ind_, minimum detectable change for an individual; MDC_group_, minimum detectable change for a group; SEM, standard error of measurement.

^a^ Includes only participants who were able to complete all three tests.

#### 3.1.2 Floor effects

No floor effects were apparent (Table 3). The number of participants who were unable or unwilling to complete the assessment increased between test 1 and test 3. By the third test, 10% of participants did not complete the test, which was due to knee pain, though one patient declined due to sciatica.

**TABLE 3:** Floor effects—number of participants who could not complete a test

	*N*	Test 1	Test 2	Test 3
Baseline	93	1 (1.1%)	2 (2.2%)	10 (10.8%)
6 months	68	5 (7.4%)	5 (7.4%)	7 (10.3%)

*Note*: One and five participants could not complete any test at baseline or 6 months respectively.

## 4 DISCUSSION

The 30 CST is a popular and recommended measure of physical function in people with knee OA. The current study is the first to assess score stability over three tests, which addresses the limitation of earlier studies which only assessed two tests. Results indicated excellent within subject reliability of a single assessor when assessed with the ICC_(2,1)_. Measurement error (SEM) between tests was approximately one stand, or 10%. When assessing change over time, an individual's score needed to change by approximately 2.5 stands (MDC_ind_), and a group's score needed to change by 0.3–0.4 stands (MDC_group_) to exceed measurement error and represent ‘real change’. No floor effects were apparent.

Previous research has shown improved performance for the 30 CST between the first and second tests (Gill & McBurney, 2008; Holm et al., 2021; Tolk et al., 2019; Unver et al., 2015), which is consistent with the current study. To assess whether performance stabilised after the second test, this study included a third test and found that performance either deteriorated a little (baseline assessment) or stabilised (6 months assessment). Performance on physical tests can be influenced by learning, motivation, pain and fatigue. Improved 30 CST performance between tests 1 and 2 could reflect learning. The size of the difference between tests 1 and 2 in the current study (0.5 to 0.8 stands) was similar in direction and magnitude to other tests in people with knee OA (Gill & McBurney, 2008; Holm et al., 2020; Tolk et al., 2019; Unver et al., 2015). Poorer performance between tests 2 and 3 at baseline could reflect reduced motivation, pain and/or fatigue. Pain is likely to have an important impact on performance in people with symptomatic knee OA; our results indicated that up to 10% of participants were unable to complete all three tests due to pain at the baseline and 6‐month assessment.

Clinicians and researchers should be aware that systematic changes in 30 CST scores can occur between repeated tests and an individual's performance might change simply as a result of repeated testing rather than due to the effects of treatment. Consistent with previous recommendations (Gill & McBurney, 2008), one warm‐up or practice test appears warranted when assessing change in individuals or single groups, but this should be weighed up against the potential for increasing pain, fatigue and floor effects. Systematic change is less influential when two or more groups are compared, because systematic change will effect both groups and it is the relative difference in performance between the two groups that will be of interest (Hopkins, 2000).

The estimates for MDC provide clinicians and researchers with information to determine if real change has occurred. Our previous research in people with advanced knee and hip OA estimated MDC_ind_ at 1.6 stands, which is approximately one stand less than the current study. Importantly, in our previous research, MDC was calculated from a group of participants who could only complete, on average, six stands, whereas in the current study it was 9–13 (SD: 2.9–5.0). When expressed as a co‐efficient of variation (i.e. measurement error as a proportion of the mean score), scores varied approximately 10% between tests in both the current and our previous study. Our previous research also combined data from knee and hip patients, which confounds comparisons to the current study. Overall, the results from these studies reiterate that measurement error for a particular test is specific to the population from which the estimates were derived. From our current and previous studies, it appears that measurement error for the 30 CST is greater in people with mild knee OA who can complete more stands than in people with advanced knee OA who can complete fewer stands.

Floor effects were not apparent for the group, based on our definition. However, a small number of participants were unable to complete any of the three tests, and some participants dropped out after the first or second test due to increased pain. Clinicians and researchers who are assessing change with the 30 CST need to be aware that the test is unable to detect deterioration in a small number of people who have poor baseline function.

### 4.1 Limitations

Results from the current study are context dependent and apply to people with a mild radiographic knee OA and assume our testing protocol is used. Changing the protocol, for example, by changing the height of the chair, is likely to affect performance and measurement error. One assessor completed all tests and her knowledge of previous test scores could have influenced subsequent scoring. Inter‐rater reliability was not determined; our intra‐rater estimates are likely to be lower than inter‐rater estimates due to greater sources of error when more than one person takes measurements (Streiner & Norman, 2008). Rater bias, where the assessor's knowledge of previous scores influences, knowingly or otherwise, the measurement of subsequent attempts, could have influenced our results (Portney & Watkins, 2015). We did not objectively assess pain, fatigue or motivation during the testing procedure and are uncertain how these factors influenced results.

## 5 CONCLUSIONS

The current study is the first, to our knowledge, to assess score stability for the 30 CST across three tests in people with knee OA, and included the largest sample size to date. Test scores demonstrated excellent intra‐session test‐retest reliability according to ICCs_(2,1)_. The estimates of MDC, which is a measure of agreement, will allow clinicians and researchers to determine if change beyond measurement error has occurred. MDC values only apply to people with mild radiographic knee OA and the use of our study protocol. Practice effects occurred between tests one and two, so when assessing change in individuals or single groups, practice effects with repeated testing might obscure or imitate changes due to interventions. A single warm up test, if the participant can tolerate it, might help reduce the influence of practice effects when assessing change over time for individuals and single groups.

## IMPLICATIONS FOR PHYSIOTHERAPY PRACTICE

- The 30 CST is a recommended performance‐based outcome measure for people with knee OA, but it's clinimetric properties have received little research attention.
- The current study is the first to assess score stability across three tests.
- Test scores demonstrated excellent test‐retest reliability with ICCs_(2,1)_ > 0.9.
- To exceed measurement error and demonstrate ‘real change’, an individual's score needs to change by at least 2.5 stands and a group's score needs to change by at least 0.3–0.4 stands.

## AUTHOR CONTRIBUTIONS

SDG conceived the study. All authors assisted with study design. RH collected the data. SDG collated and analysed the data. SDG wrote the first draft of the manuscript. All authors reviewed and approved the final version of the manuscript.

## CONFLICT OF INTEREST

Funding was received by the Royal Australia and New Zealand College of Radiologists to complete a randomised controlled study in which some participants in the current study were enrolled.

## ETHICS STATEMENT

The study was approved by the organisation's Human Research Ethics Committee (refs 15/101 and 18/135) and the clinical trial associated with the current study was prospectively registered with the ANZCTR (ACTRN12616000770460).

## Data Availability

The data that support the findings of this study are available from the corresponding author upon reasonable request.

## Protocol for the 30 s chair stand test

**Set‐up:**

1. Use a chair with a 17‐inch seat height.
2. Place the chair against the wall to prevent it from moving during the test.
3. Two lines are placed on the seat 10 and 14 inches from the front edge of the chair.
4. Flat or low‐heeled shoes must be worn. If the patient has high‐heeled shoes of greater than one inch, ask them to perform the test barefooted.
5. Sit the patient in the middle of the chair so that their sacrum is somewhere between the two lines at a place the patient finds comfortable.
6. Allow the patients to position their feet as they find comfortable.
7. Do not allow them to lean against the backrest of the chair but ask them to sit up with their back straight.
8. Ask them to cross their arms across their chest.

**Instructions to participant:**

1. ‘This test looks at how many times you can stand and sit from a chair in 30 s’.
2. ‘If you do not fully stand or sit down so that your bottom touches the seat, that repetition will not be counted’.
3. Demonstrate the movement.
4. Allow one practice stand by the patient.
5. ‘When I say “GO”, I want you to stand up and sit down as many times as you can in 30 s’.
6. ‘If you have pain that becomes too uncomfortable, you are allowed to stop the test’.
7. ‘Do you understand what you need to do?’.
8. ‘Are you ready?’.
9. ‘Ready, set, GO’.

**General guidelines**

1. If the patient is more than halfway up at the end of 30 s, it counts as a full stand.
2. Do not offer any encouragement before or after each test other than ‘well done’.
3. If a patient expresses concern about performing a task, tell him/her to ‘do the best that you can’.
